# Risk factors of dysphagia in patients with ischemic stroke: A meta-analysis and systematic review

**DOI:** 10.1371/journal.pone.0270096

**Published:** 2022-06-16

**Authors:** Cui Yang, Yun Pan

**Affiliations:** 1 Department of Neurology, The First People’s Hospital of Lianyungang, The Affiliated Lianyungang Hospital of Xuzhou Medical University, Jiangsu, China; 2 Division of Rheumatology, The First People’s Hospital of Lianyungang, The Affiliated Lianyungang Hospital of Xuzhou Medical University, Jiangsu, China; Universita degli Studi di Roma La Sapienza, ITALY

## Abstract

**Background:**

Dysphagia is a common yet serious complication in stroke patients. We aimed to conduct a meta-analysis and systematic review to evaluate the risk factors of dysphagia in patients with ischemic stroke, to provide insights to the clinical treatment and nursing care of dysphagia.

**Methods:**

We searched PubMed, Embase, Cochrane Library, Web of Science, China National Knowledge Infrastructure (CNKI) and Wanfang Database, China Biomedical Literature Database (CBM) for studies on dysphagia in patients with ischemic stroke up to January 31, 2022. The quality of the literature was evaluated using the Newcastle-Ottawa scale. Meta-analysis was performed using RevMan 5.3 software.

**Results:**

A total of 10 studies involving 4637 ischemic stroke patients were included, 1183(25.51%) patients had dysphagia after stroke. The synthesized outcomes showed that elder age (SMD = 0.42, 95%CI:0.34–0.50), hypertension (OR = 1.96, 95%CI:1.48–2.61), diabetes (OR = 1.83, 95%CI:1.47–2.28), brainstem stroke (OR = 2.12, 95%CI:1.45–3.09) were associated with dysphagia in patients with ischemic stroke (all P<0.05). There was no significant difference in the gender between dysphagia and no dysphagia patients (OR = 1.07, 95%CI:0.91–1.27, P = 0.40). Egger regression tests indicated there were no significant publication biases in the synthesized outcomes (all P>0.05).

**Conclusions:**

Elder age, hypertension, diabetes and brainstem stroke are associated with the development of dysphagia in patients with ischemic stroke. Attention should be paid to the assessment and early intervention of those risk factors for dysphagia to improve the prognosis of stroke patients.

## Background

Stroke has become the leading cause of death in the world, of which ischemic stroke is the most common type of stroke, accounting for about 80% stroke [1, 2]. Dysphagia is one of the common complications after stroke, the incidence of dysphagia in acute stroke patients is 34.11%-80.05% [3, 4]. Dysphagia can significantly increase the risk of aspiration, pneumonia, and prolong hospital stay in stroke patients [5, 6]. Additionally, dysphagia is an independent risk factor for malnutrition, poor functional recovery, and post-stroke depression [7]. Therefore, the prevention and treatment of dysphagia after stroke is of great significance to the quality of life and prognosis of stroke patients.

Identifying risk factors for dysphagia is critical for the prevention and treatment of dysphagia after stroke. Previous studies [8, 9] have pointed out that the occurrence of dysphagia in stroke patients is affected by many factors. Among them, age, history of cerebrovascular disease, infarction site and other important factors that may affect the occurrence of dysphagia. Besides, the patient gender and muscle strength, etc. may also affect the occurrence of dysphagia, but research results are inconsistent. At present, for the investigations and researches on dysphagia in patients with ischemic stroke, due to the differences in study population, the screening and diagnosis methods of dysphagia, the sample size and other factors, the results are also quite different. Therefore, this meta-analysis aimed to conduct a data synthesis analysis and systematic review of the current risk factors for dysphagia in ischemic stroke patients, to evaluate the risk factors for dysphagia in ischemic stroke patients, to provide evidence support for the clinical prevention, treatment and nursing care of dysphagia.

## Methods

Ethical approval was not necessary since our study was a meta-analysis and systematic review. This meta-analysis was conducted and reported according to the guidelines for preferred reporting items for systematic reviews and meta-analyses (PRISMA statement) [10]. In this study, all methods were performed in accordance with the relevant guidelines and regulations.

### Inclusion and exclusion criteria

The inclusion criteria for this meta-analysis were as follows: (1) The study population were patients with ischemic stroke diagnosed by head CT or MRI, and the patients were aged ≥18 years; (2) The swallowing function assessment tool was used to determine whether the patients had dysphagia. (3) cohort study or case-control study design. (4) The publication language of the article was limited to Chinese and English. The literature exclusion criteria for this meta-analysis were as follows: (1) patients with non-ischemic stroke; (2) duplicate publications; (3) studies with incomplete information or data for extraction; (4) literature with low quality.

### Literature search

The two authors searched PubMed, Embase, Cochrane Library, Web of Science, China National Knowledge Infrastructure (CNKI) and Wanfang Database, China Biomedical Literature Database (CBM) for studies on dysphagia in patients with ischemic stroke. The search time limit of each database was from the establishment of the database to January 31, 2022. We combined subject terms and free terms to conduct the literature search. The search terms used in this meta-analysis were (“stroke” OR “ischemic stroke” OR “brain ischemia” OR “cerebral infarction” OR “intracranial embolism” OR “cerebral embolism”) AND (“’swallowing disorder” OR “swallowing dysfunction” OR "dysphagia").

### Literature screening and data extraction

Two authors independently searched the literature according to the retrieval strategy. We initially screened the literature according to the title and abstract, and then we read the full text for re-screening and cross-checking. If there was any disagreement, it would be resolved through negotiation. The contents of the literature data extracted in this meta-analysis included the first author, study site, sample size, number of cases of dysphagia, details of dysphagia assessment methods (tools, evaluators, assessment timepoint), related risk factors for dysphagia, and research conclusions.

### Methodological quality assessment

Two authors independently used the Newcastle-Ottawa scale (NOS) [11] to evaluate the quality of included studies. If any disagreements occurred during the evaluation process, they were resolved through discussion. The NOS scale evaluated the quality of the literature in three aspects: patient selection (4 items, 4 points), comparability (1 item, 2 points), and exposure assessment (3 items, 3 points). The total NOS score is 9 points, and literatures with NOS ≥ 7 points are generally considered as high-quality.

### Data analysis

RevMan 5.3 software was used for statistical analyses in this meta-analysis. Binary outcomes were presented as Mantel–Haenszel-style odds ratios (OR) with 95% confidence intervals (CI). Continuous outcomes were presented as standardized mean differences (SMD). Fixed-effect model was selected in cases of homogeneity (P value of χ2 test >0.10 and I^2^ < 50%), and random-effect model was used in cases of obvious heterogeneity (P value of χ2 test >0.10 and I^2^≥50%). Publication bias were evaluated by using funnel plots, and asymmetry was assessed with Egger regression test. In this meta-analysis, P<0.05 was considered that the difference was statistically significant between groups.

## Results

### Study inclusion

**Fig 1:**
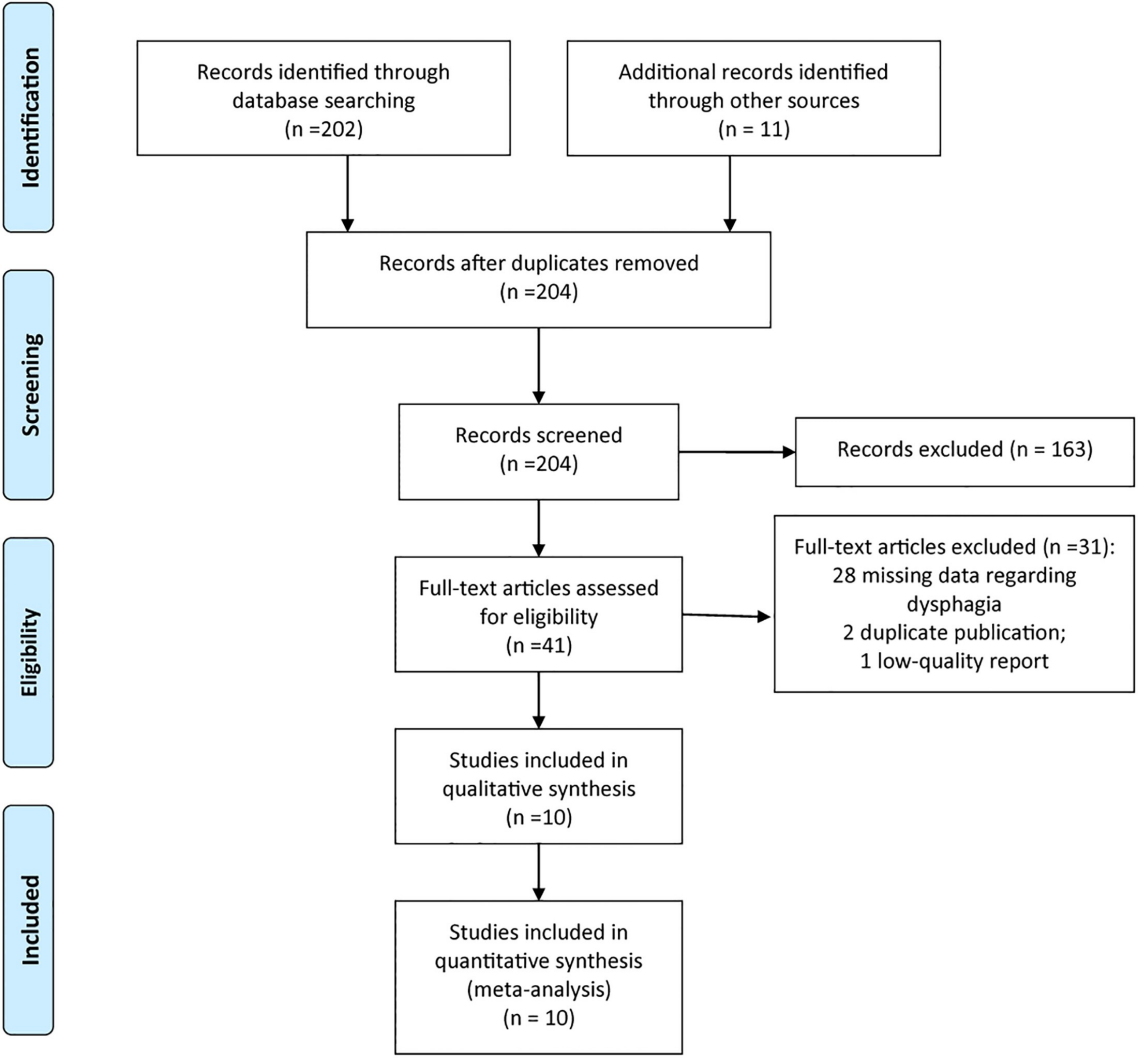
The flow chart of study selection.

The process of selecting studies is presented in Fig 1. The first search identified 213 potentially relevant reports. Of those identified articles, 9 reports were excluded as duplicates. After viewing the titles and abstracts of the 204 remaining reports, the full texts of 41 reports were retrieved. Among them, 31 studies were excluded with failure to meet criteria. Finally, 10 studies [12–21] were included in this present meta-analysis.

### The characteristics of included studies

Of the 10 included studies [12–21], a total of 4637 ischemic stroke patients were involved, and 1183(25.51%) patients had dysphagia after stroke. As presented in Table 1, Of the 10 included studies [12–21], 4 studies were assessed for dysphagia within 24 hours of admission. The dysphagia assessment tools used in the studies mainly included standardized swallowing assessment (SSA), water swallowing test(WST), Burke Dysphagia Screening Test(BDST), video fluoroscopy swallowing study (VFSS), fiberoptic endoscopic evaluation of swallowing(FEES). The influencing factors analyzed in the 10 reports [12–21] mainly included the general demographic data, underlying diseases, disease severity, and stroke site of patients.

**Table 1:** The characteristics of included studies.

Study	Country	Sample size	Cases of dysphagia	Swallowing disorder assessment tool	Dysphagia evaluator	Timepoint of dysphagia assessment
Beharry 2019	New Zealand	340	81	BDST	Nurse	24 hours after admission to hospital
Cong 2012	China	496	103	WST	Physician	At the admission to hospital
Flowers 2017	Canada	160	76	Clinical symptom and tool assessment	SLP	Within 14 days after stroke
Hao 2018	China	177	87	SSA	Rehabilitation physician	24 hours after admission to hospital
Henke 2017	Germany	1442	413	Two-step assessment of swallowing function	SLP	24 hours after admission to hospital
Huang 2007	China	563	75	Swallowing disorders clinical screening system	Physician	During the hospital stay
Lapa 2017	Germany	59	14	Clinical swallowing disorder assessment and FEES	SLP	24 hours after admission to hospital
Meng 2021	China	542	202	WST	Rehabilitation technician and stroke rehabilitation specialist nurse	48 hours after admission to hospital
Remesso 2011	Brazil	596	117	Bedside symptom assessment and tool assessment	Medical team	Within 14 days after admission to hospital
Yang 2018	South Korea	262	15	VFSS	Rehabilitation physician	During the hospital stay

Notes: SSA, standardized swallowing assessment; WST, water swallowing test; BDST, Burke Dysphagia Screening Test; VFSS, video fluoroscopy swallowing study; FEES, fiberoptic endoscopic evaluation of swallowing.

### The quality of included studies

As indicated in Table 2, the NOS score of 3 papers was rated as 8 points, and 7 papers were rated as 7 points, the quality of the 10 included articles was generally high.

**Table 2:** The NOS score of included studies.

Study	Patient selection	Comparability	Exposure assessment	NOS total score
Beharry 2019	3	2	3	8
Cong 2012	3	2	2	7
Flowers 2017	3	2	2	7
Hao 2018	3	2	3	8
Henke 2017	3	2	2	7
Huang 2007	3	2	2	7
Lapa 2017	3	2	3	8
Meng 2021	3	2	2	7
Remesso 2011	3	2	2	7
Yang 2018	3	1	3	7

### Meta-analysis

**Fig 2:**
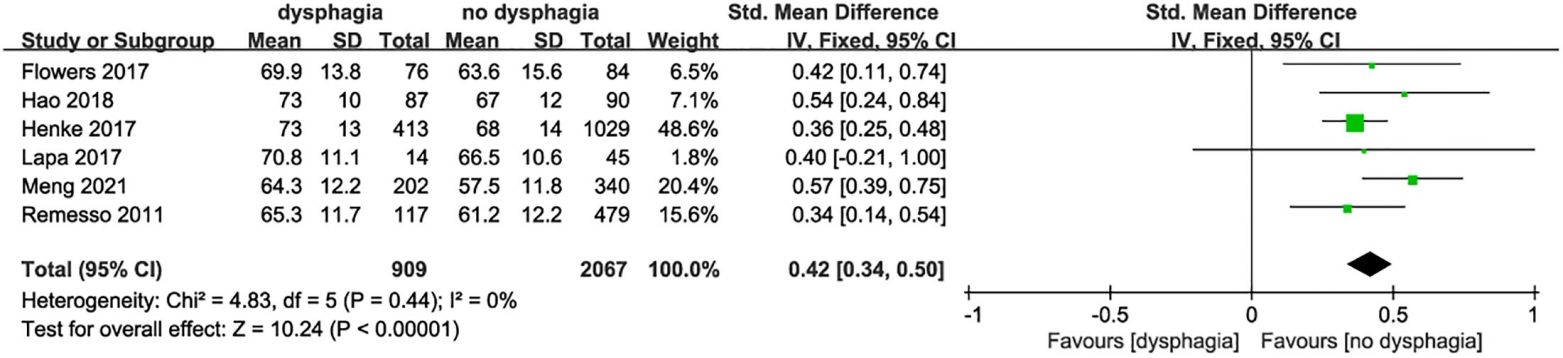
Forest plot for the relationship between age and dysphagia after stroke.

*Age and dysphagia after stroke* 6 studies [12, 15, 16, 18–20] evaluated the relationship between age and dysphagia, and there was no statistical heterogeneity among the studies (I^2^ = 0, P = 0.44). The fixed effect model was used for statistical analysis. The results showed that the risk of dysphagia was higher in stroke patients with elder age (SMD = 0.42, 95%CI:0.34–0.50, P<0.001, Fig 2).

**Fig 3:**
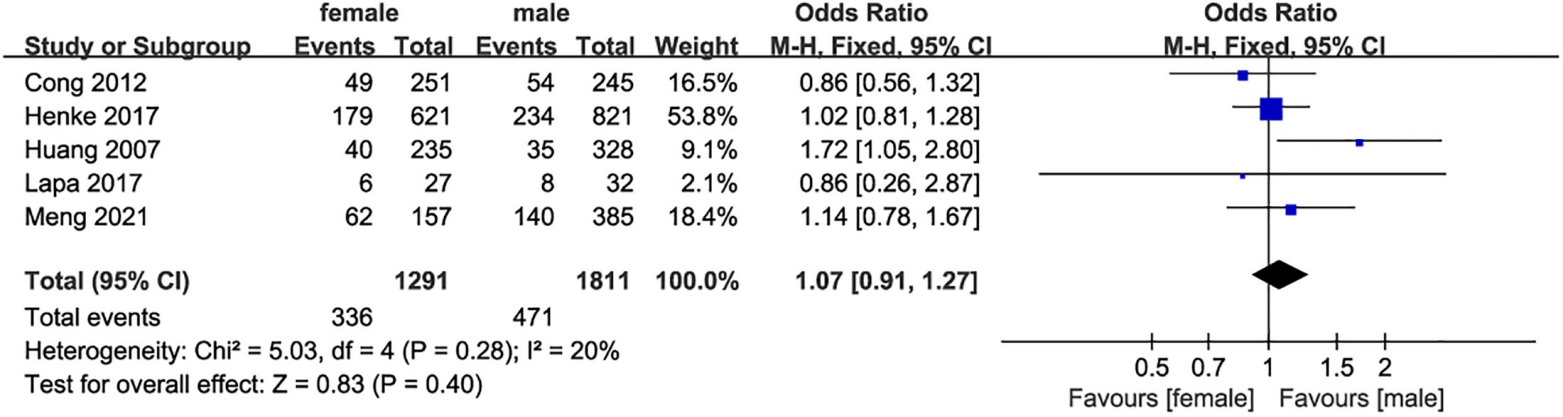
Forest plot for the relationship between gender and dysphagia after stroke.

*Gender and dysphagia after stroke* 5 studies [14, 16–19] evaluated the relationship between gender and dysphagia, and there was no statistical heterogeneity among the studies (I^2^ = 20, P = 0.28). The fixed effect model was used for statistical analysis. The results showed that the there was no significant difference in the gender between dysphagia and no dysphagia patients(OR = 1.07, 95%CI:0.91–1.27, P = 0.40, Fig 3).

**Fig 4:**
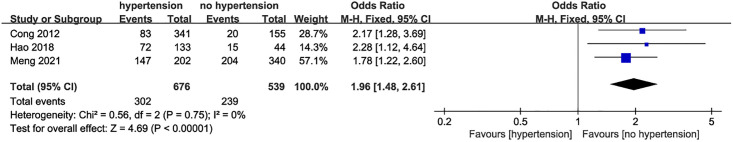
Forest plot for the relationship between hypertension and dysphagia after stroke.

*Hypertension and dysphagia after stroke* 3 studies [12, 14, 19] evaluated the relationship between hypertension and dysphagia, and there was no statistical heterogeneity among the studies (I^2^ = 0, P = 0.75). The fixed effect model was used for statistical analysis. The results showed that the risk of dysphagia was higher in stroke patients with hypertension (OR = 1.96, 95%CI:1.48–2.61, P<0.001, Fig 4).

**Fig 5:**
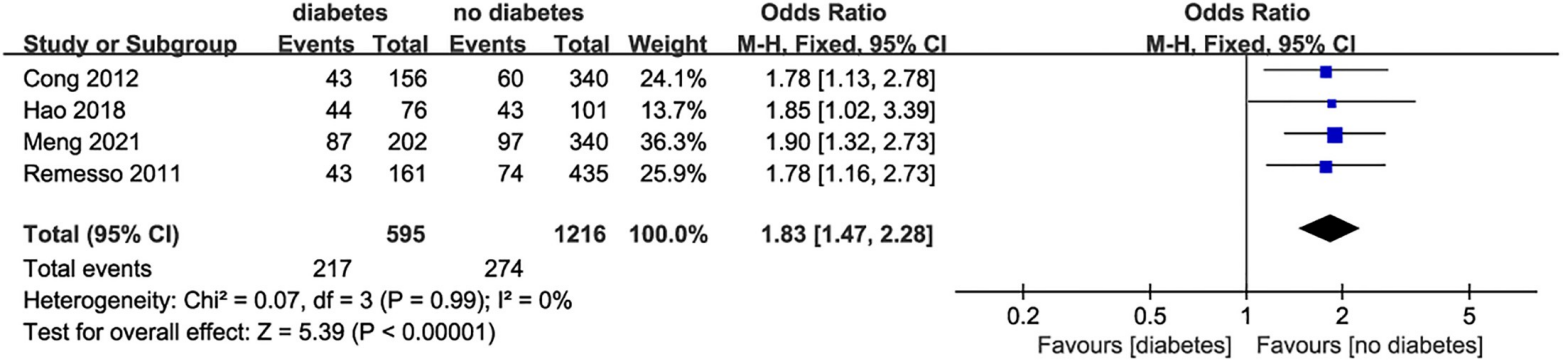
Forest plot for the relationship between diabetes and dysphagia after stroke.

*Diabetes and dysphagia after stroke* 4 studies [12, 14, 19, 20] evaluated the relationship between diabetes and dysphagia, and there was no statistical heterogeneity among the studies (I^2^ = 0, P = 0.99). The fixed effect model was used for statistical analysis. The results showed that the risk of dysphagia was higher in stroke patients with diabetes (OR = 1.83, 95%CI:1.47–2.28, P<0.001, Fig 5).

**Fig 6:**
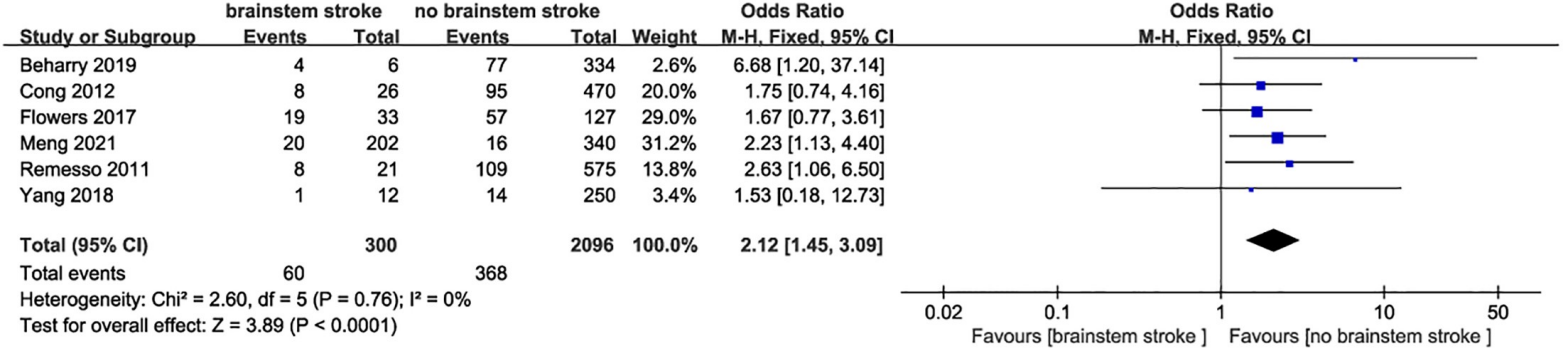
Forest plot for the relationship between brainstem stroke and dysphagia after stroke.

*Brainstem stroke and dysphagia after stroke* 6 studies [13–15, 19–21] evaluated the relationship between stroke site and dysphagia, and there was no statistical heterogeneity among the studies (I^2^ = 0, P = 0.76). The fixed effect model was used for statistical analysis. The results showed that the risk of dysphagia was higher in patients with brainstem stroke (OR = 2.12, 95%CI:1.45–3.09, P<0.001, Fig 6).

### Publication of bias and sensitivity analyses

**Fig 7:**
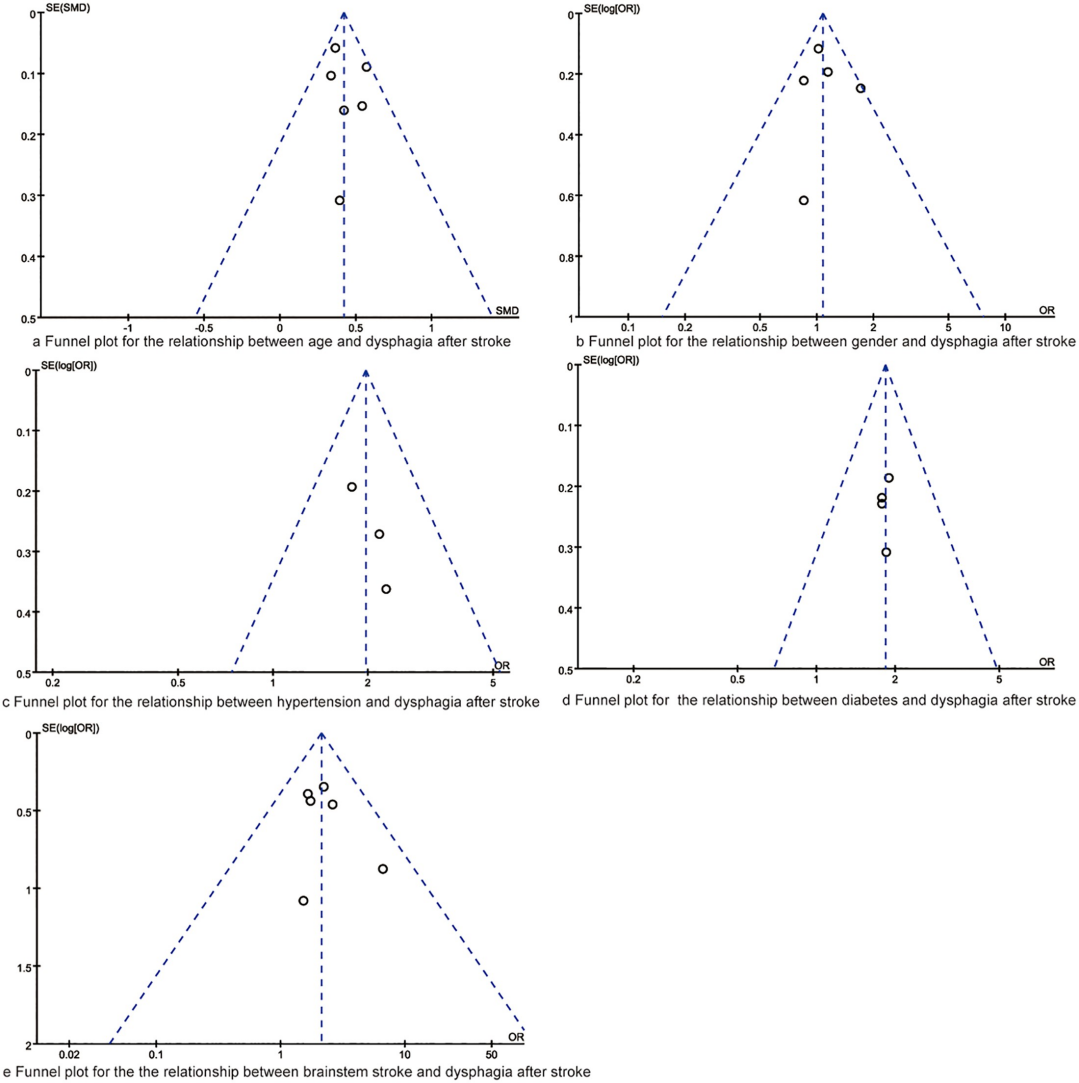
Funnel plots for the synthesized outcomes.

The publication of bias was evaluated with funnel plot and Egger regression test. As indicated in Fig 7, the dots in the funnel plot of synthesized outcomes were evenly distributed, and results of Egger regression test indicated there were no significant publication biases in the synthesized outcomes (all P>0.05).

Sensitivity analyses, which evaluated the impact of one single study on the overall risk estimated by removing one study in each turn, suggested that the overall risk estimates were not substantially changed by any one study.

## Discussions

Stroke is a common and frequently-occurring disease of the nervous system, and it is an important cause of death or disability of residents worldwide, and the recurrence rate is high [22, 23]. According to statistics [24, 25], there are about 2 million new stroke patients in China every year, of which 22%-65% of stroke patients are accompanied by dysphagia, the incidence of dysphagia within 3 days of stroke patients is 12%-67%. Aspiration occurs in 10% of patients with dysphagia, and 33% of patients with dysphagia can develop pneumonia, which significantly increased the risk of death [26]. However, many screenings especially bedside assessment evaluation (BSE) are focused on dysphagia without considering aspiration and vice-versa. It’s been reported that dysphagia may occur without aspiration [27]. Conversely, high sensitive BSEs designed to detect also aspiration and tested against the FEES [28–30] are more likely to depict the real situation, thus being more useful to design studies on post-stroke aspiration prevention. Therefore, in clinical work, it is important to identify the risk factors for dysphagia to prevent and treat dysphagia after stroke. The results of this meta-analysis suggest that age, hypertension, diabetes, and brainstem stroke are independent risk factors for dysphagia in patients with ischemic stroke, early targeted prevention and nursing care are needed for those patients in clinical settings.

Previous studies [31–33] have pointed out that dysphagia after stroke is mainly caused by damage to the swallowing cortex center, cortical descending fibers, medulla oblongata swallowing center and extrapyramidal system. The physiological process of dysphagia includes cognitive and psychological disorders, organic lesions and functional abnormalities. The primary lesions of dysphagia after stroke are located in both cerebral cortex or brainstem tracts [34]. Swallowing disorders can occur in all stages from the cognitive stage to the esophageal stage [35]. Patients will experience prolonged oral passage time and pharyngeal swallowing delay, and they are prone to aspiration before and during swallowing [36]. Treatment and nursing care of patients with dysphagia after stroke is a holistic process from swallowing assessment to rehabilitation, emphasizing multidisciplinary participation and comprehensive training [37–39]. The assessment of dysphagia after stroke should be based on the clinical manifestations of patients, and fully consider the advantages of assessment methods, so as to improve the accuracy and effectiveness of dysphagia screening and treatment [40–42].

Age is an important factor affecting patients with dysphagia after stroke. Previous studies [43, 44] have shown that age is a risk factor for dysphagia, and with age increases, the risk of dysphagia in stroke patients also increases, which is consistent with our findings. The possible reasons may be related to the gradual weakening of various body functions as the patient aging, and the weakening of the body function can further lead to the dysfunction of the patient’s oral and maxillomandibular system (chewing, swallowing, breathing, and vocalization), thereby causing dysphagia [45, 46]. In addition, older patients have a higher probability of degeneration of advanced cranial nerve function and abnormal swallowing reflex function, which further increases the susceptibility of swallowing dysfunction [27, 47–49]. Due to the weakening of the transport capacity of the tongue muscle and the collapse of the tongue muscle in elderly patients, the food bolus will leak in advance and later, and the oral transport time will be prolonged during the feeding process [50, 51]. Clinical medical workers should fully evaluate the swallowing function of elderly stroke patients with dysphagia, and carry out targeted rehabilitation training to prevent the occurrence of aspiration.

Hypertension and diabetes are not only the risk factors for stroke, but also the risk factors for dysphagia in stroke patients [52, 53]. The results of this study have showed that the incidence of dysphagia in ischemic stroke patients with hypertension and diabetes is significantly higher than that of patients without hypertension and diabetes, which may have a joint effect with chronic diseases affecting the overall function of patients, thereby increasing the risk of dysphagia. Brainstem lesions can affect the sensitivity of the tongue and cheeks, and it may cause swallowing and laryngeal muscle dysfunction, which are the independent risk factors for swallowing dysfunction. It’s been reported that patients with brainstem lesions are often accompanied by covert aspiration, leading to aspiration pneumonia [54–56]. The results of this meta-analysis have showed that brainstem infarction is a risk factor for dysphagia in patients with ischemic stroke, which may be related to the existence of cranial nerve nuclei in the medulla oblongata that control and regulate the swallowing reflex [57]. We have not found the gender differences in the occurrence of dysphagia after stroke. However, previous studies [58, 59] have reported that men are at greater risk of dysphagia and pneumonia after stroke than women. Therefore, the gender difference in the occurrence of dysphagia after stroke remains to be further studied in the future.

It must be noted that for silent aspiration, whose detection is usually failed from most BSEs, and that could be of particular relevance for pneumonia after stroke. In fact, by focusing on the overt sign of aspiration to diagnose post-stroke dysphagia, such as cough or voice change, the silent aspiration can be undiagnosed, with a relevant increase in relative risk of pneumonia and poorer stroke clinical outcome. It’s been reported that although advances have been made in dysphagia care, prevalent screening and treatment practices remain insufficient to reduce pneumonia and decrease case fatality in dysphagic stroke patients [50]. Besides, in stroke patients who passed low-sensitive screening for dysphagia compared to those who passed high-sensitive ones who can detect also silent aspiration [60]. The stroke severity, as assessed with the NIHSS, dramatically affects the incidence and severity of post-stroke dysphagia. NIHSS is an independent risk factor for swallowing impairment after stroke. Moreover, it is very relevant that an NIHSS ≥ 12 has been suggested as the cut-off value to predict, upon admission, those stroke patients who will probably remain dysphagic after 14 days follow-up [16, 28]. Thus, identifying dysphagic patients through a highly accurate screening tool might be crucial in reducing the complications after stroke.

There are certain limitations of this meta-analysis worth considering. First, the studies included in the meta-analysis were all derived from published literature, and gray literature was not included, which may have potential publication bias. Second, there are certain differences in swallowing function assessment time, swallowing function assessment screening tools and risk measurement, etc., which may affect the study results even though we did not find the heterogeneity amongst the synthesized outcomes. Third, we only focused on the population with ischemic stroke, the choice to exclude other types of stroke could be reasonable for increasing the certitude of the results but has also the weakness of limiting the generalizability of results. Finally, the data reported for some other potentially influencing factors in the included studies are very limited. Due to the inconsistent data types of NIHSS scores in the literature included in this meta-analysis, some studies were continuous variables, some studies were categorical variables, and the classification thresholds of NIHSS scores were different, so this study could not analyze the effect of NIHSS scores on dysphagia in stroke patients. Some studies have shown that NIHSS score and muscle strength are independent predictors of dysphagia in patients with acute stroke. The association of these factors with dysphagia after stroke requires further analysis and investigation in the future.

## Conclusions

In conclusion, this meta-analysis has found that age, hypertension, diabetes, and brainstem stroke are the independent risk factors for dysphagia in patients with ischemic stroke. In clinical work, high-risk patients for dysphagia in patients with ischemic stroke should be vigilant, comprehensive and systematic evaluation should be carried out as soon as possible, and early intervention and early nursing care should be taken for patients with changes in swallowing function, so as to improve the quality of life of patients.

## Abbreviations

BDST: Burke Dysphagia Screening Test
BSE: bedside assessment evaluation
CI: confidence intervals
CNKI: China National Knowledge Infrastructure
FEES: fiberoptic endoscopic evaluation of swallowing
NOS: Newcastle-Ottawa scale
OR: odds ratios
PRISMA: preferred reporting items for systematic reviews and meta-analyses
SMD: standardized mean differences
SSA: standardized swallowing assessment
VFSS: video fluoroscopy swallowing study
WST: water swallowing test
